# Regulating arachidonic acid metabolism: a novel strategy to prevent colorectal inflammatory cancer transformation

**DOI:** 10.7150/jca.118694

**Published:** 2025-10-01

**Authors:** Sisi Ren, Lu Lu, Hang Su, Zongping Li, Sumei Li, Jiashu Pan, Yujing Liu, Guang Ji, Hanchen Xu

**Affiliations:** 1 Institute of Digestive Diseases, Longhua Hospital, China-Canada Center of Research for Digestive Diseases (ccCRDD), Shanghai University of Traditional Chinese Medicine, Shanghai, 200032, China.; 2 State Key Laboratory of Integration and Innovation of Classic Formula and Modern Chinese Medicine, Shanghai University of Traditional Chinese Medicine, Shanghai, 200032, China.; 3 Shanghai Frontier Research Center of Disease and Syndrome Biology of Inflammatory Cancer Transformation, Shanghai, 200032, China.

**Keywords:** colorectal cancer, arachidonic acid, gut microbiota, inflammatory cancer transformation, cancer therapy

## Abstract

Colorectal cancer (CRC) ranks among the leading causes of cancer-related morbidity and mortality worldwide, with colitis-associated colorectal cancer (CAC) driven by inflammatory cancer transformation. Arachidonic acid (AA), a key ω-6 polyunsaturated fatty acid, and its metabolites, including prostaglandins (PGs) and leukotrienes (LTs), play pivotal roles in this process by modulating inflammation, immune responses, and the intestinal microenvironment. Notably, a multi-enzyme co-expression nanoplatform integrating lipoxygenase (LOX) and phospholipase A_2_ (PLA_2_) has been developed, synergistically inducing immunogenic ferroptosis and upregulating AA expression to enhance CD8^+^ T cell-mediated anti-tumor immunity. Additionally, dual COX-2/soluble epoxide hydrolase (sEH) inhibitors, such as PTUPB, demonstrate enhanced anti-tumor activity and reduced toxicity when combined with cisplatin, offering a promising approach to mitigate gastrointestinal side effects of nonsteroidal anti-inflammatory drugs (NSAIDs). Furthermore, natural products like ginsenoside Rk3 and berberine have been identified to regulate AA metabolism and gut microbiota, alleviating CAC by modulating lipid peroxidation and inflammatory pathways. This review synthesizes these innovative findings, highlighting the role of AA metabolism in maintaining intestinal homeostasis, promoting inflammatory cancer transformation, and serving as a therapeutic target to inhibit CAC progression, thus providing new insights into its prevention and treatment.

## 1. Introduction

Colorectal cancer (CRC) is one of the most common malignant tumors of the digestive system 1. According to the latest statistics from the National Cancer Center (NCC), CRC has the second highest incidence rate and the fourth highest mortality rate among all malignant tumors, and the incidence rate continues to increase 2. Colitis-associated colorectal cancer (CAC) refers to CRC arising from chronic intestinal inflammation, primarily in ulcerative colitis (UC) and Crohn's disease (CD) patients. Clinically, CAC is characterized by earlier disease onset, multifocal lesions, and a higher likelihood of aggressive tumor behavior compared to sporadic CRC. The inevitable consequence of CAC is the progression from chronic inflammation to dysplasia and malignancy, driven by persistent epithelial injury, immune dysregulation, and microbial dysbiosis, with potential outcomes including increased metastasis and reduced survival 3.

Polyunsaturated fatty acids (PUFAs) are essential fatty acids that may play a potential role in regulating inflammation, particularly in the pro-cancer inflammatory milieu of the colon. The two main types of PUFAs are omega-3 (ω-3) fatty acids and omega-6 (ω-6) fatty acids. A systematic review found that a high dietary intake of ω-3 fatty acids reduced the risk of CRC, and that the risk was higher with a high dietary ω-6/ω-3 ratio 4. Furthermore, statistical analyses found that elevated hereditary PUFAs were strongly associated with CRC and emphasized the high expression of ω-6 as a potential mediator 5.

Arachidonic acid (AA) is one of the ω-6 fatty acids and one of the most abundant and widely distributed PUFAs in mammals. AA can be converted to various metabolites in the body, most of which have potent physiological effects and a wide range of actions and are important for cellular regulation. AA and its metabolites regulate inflammatory responses critical to CAC onset and progression 6, 7. Given its wide range and importance, the functional study of AA metabolic pathways and metabolites has been highly valued by the life science and medical communities, and the present review will systematically elucidate the mechanism of AA and its metabolism in the inflammatory cancer transformation of CAC.

## 2. Metabolic pathways of arachidonic acid

**Figure 1:**
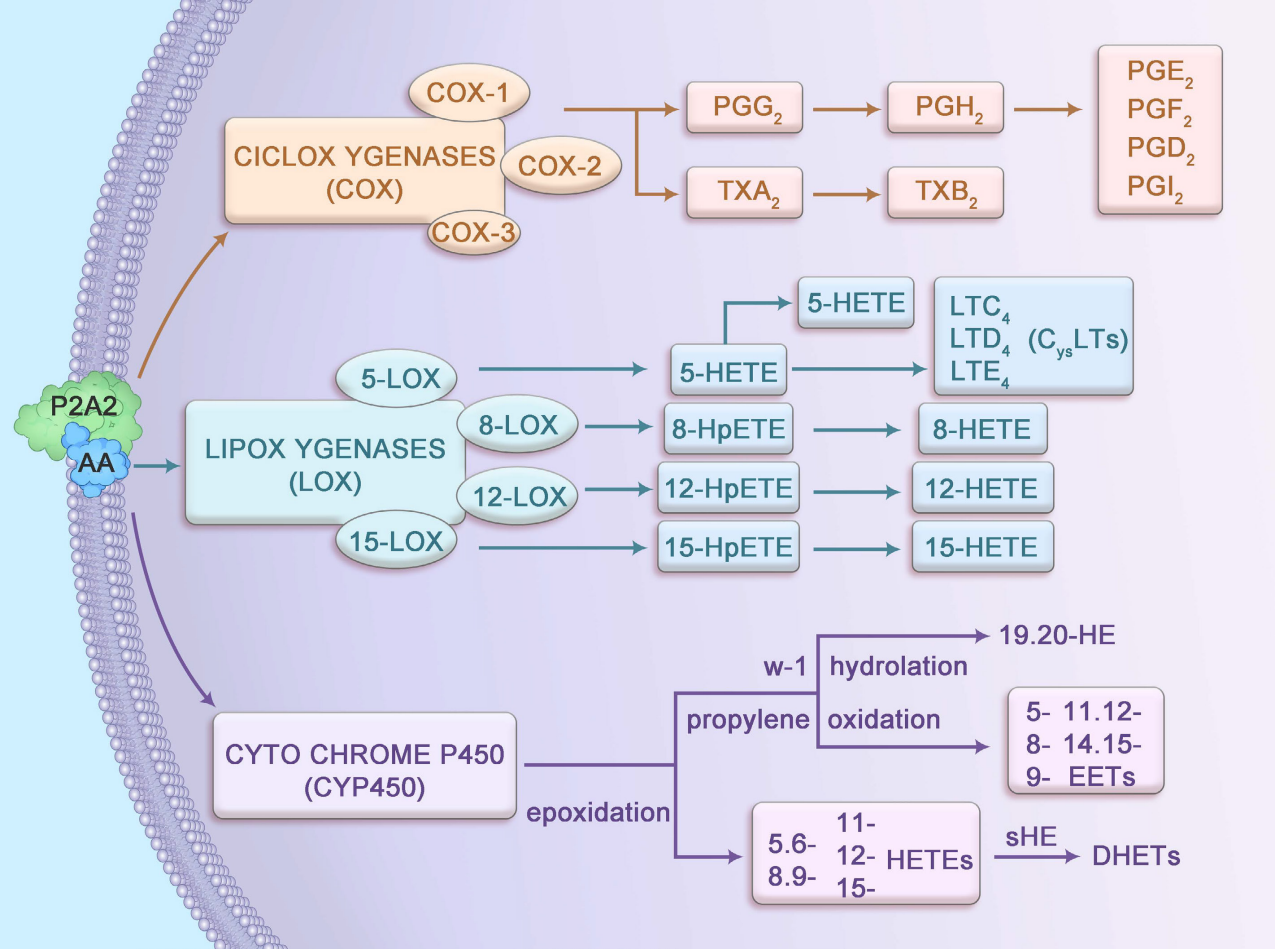
Metabolic pathways of arachidonic acid.

AA, a 20-carbon ω-6 polyunsaturated fatty acid (20:4n-6), possesses four cis-double bonds at positions 5, 8, 11, and 14, conferring high flexibility and reactivity that facilitate its role as a substrate for enzymatic metabolism. Stored primarily as an esterified component of membrane phospholipids, AA is released by cytosolic phospholipase A_2_ (cPLA_2_), which is activated by calcium-dependent translocation to the membrane in response to inflammatory stimuli. The liberated AA undergoes metabolism via three primary pathways: cyclooxygenase (COX), lipoxygenase (LOX), and cytochrome P450 (CYP450), each catalyzed by enzymes with distinct kinetic properties (Figure 1). For instance, COX-2 exhibits a higher affinity for AA compared to COX-1, enabling rapid production of prostaglandin H_2_ (PGH_2_) under inflammatory conditions. Similarly, 5-LOX, activated by 5-lipoxygenase-activating protein (FLAP), converts AA into 5-hydroperoxyeicosatetraenoic acid (5-HPETE) with high specificity, subsequently forming leukotriene B_4_ (LTB_4_). These metabolites interact with G-protein-coupled receptors (such as EP1-4 for PGE_2_, BLT_1_ for LTB_4_), triggering downstream signaling cascades such as cAMP/PKA and NF-κB, which are critical in CAC pathogenesis 8, 9.

The COX pathway comprises three isoforms: COX-1, COX-2, and COX-3, each with distinct expression patterns and functions. COX-1, constitutively expressed across most tissues, supports physiological processes such as promoting intestinal epithelial cell (IEC) proliferation and enhancing digestive juice secretion 10. PGs produced by COX-1 maintain gastrointestinal and tissue homeostasis 11 and synergize with enzymes to regulate biological processes, including apoptosis and cell cycle progression 12. In contrast, COX-2 is inducible, primarily expressed in response to inflammatory stimuli, and is rarely present in resting cells 13. COX-3 is predominantly found in the cerebral cortex and heart, with its role less clearly defined. In the presence of COX enzymes, AA is converted into PGG_2_ and PGH_2_, and occasionally thromboxane A_2_ (TXA_2_), which has a short half-life and is rapidly converted to stable TXB_2_. PGG_2_ and PGH_2_ are further transformed by isomerases into prostaglandins such as PGD_2_, PGF_2_, PGE_2_, and PGI_2_, which mediate inflammatory and homeostatic responses.

The LOX pathway involves four key enzymes—5-LOX, 8-LOX, 12-LOX, and 15-LOX—that metabolize AA into bioactive lipid mediators. The 5-LOX enzyme, activated by FLAP, is the primary producer of leukotrienes (LTs), which regulate both normal homeostasis and inflammatory responses 14. LTs are categorized into LTB_4_, a chemokine, and cysteinyl leukotrienes (LTC_4_, LTD_4_ and LTE_4_). LTB_4_ drives neutrophil recruitment, vascular leakage, and epithelial barrier function, while LTC_4_ and LTD_4_ modulate IEC proliferation and survival through effects on vascular permeability. LTE_4_ serves as a clinical biomarker for asthma triggers 15, 16. Additionally, 8-LOX, 12-LOX, and 15-LOX convert AA into 8-HPETE, 12-HPETE, and 15-HPETE, respectively, which are subsequently dehydrated to form 8-HETE, 12-HETE, and 15-HETE, contributing to inflammatory signaling.

In the CYP450 pathway, AA undergoes epoxidation to produce 5,6-, 8,9-, 11,12-, and 14,15-epoxyeicosatrienoic acids (EETs), which are hydrolyzed by soluble epoxide hydrolase (sEH) into biologically inactive dihydroxyeicosatrienoic acids (DHETs). Additionally, AA is metabolized via propylene oxidation to yield 5-, 8-, 9-, 11-, 12-, and 15-hydroxyeicosatetraenoic acids (HETEs) and via ω-1 hydroxylation to produce 19- and 20-HETEs. These metabolites regulate vascular tone, inflammation, and cellular signaling, with emerging roles in the inflammatory microenvironment of CAC.

## 3. Arachidonic acid metabolism is involved in intestinal inflammation and tumorigenesis

The etiology of inflammatory bowel disease (IBD), encompassing UC and CD, remains multifactorial, involving genetic, environmental, and microbial factors. Central to IBD pathogenesis is chronic intestinal inflammation, characterized by epithelial damage and leukocyte infiltration, which is closely linked to the activation of AA metabolic pathways. Elevated AA levels have been observed in the inflamed mucosa of UC patients, with concentrations correlating strongly with the severity of inflammation 17. Preclinical studies demonstrate that oral AA administration exacerbates inflammation in IBD mouse models, upregulating COX-2 and LTB_4_ expression, while exerting no significant effect in healthy controls 18. AA-derived metabolites, such as eicosanoids, activate transient receptor potential vanilloid 4, a calcium channel, leading to increased intracellular calcium and chemokine release, thereby amplifying IBD-associated inflammation 19. Notably, PGE_2_, a downstream AA metabolite, promotes Th17 cell-mediated inflammatory responses, further driving disease progression 20. Clinical studies in adolescent IBD patients reveal significantly elevated levels of TXB_2_, LTB_4_, and 9S-HODE during active disease phases compared to remission, with 15S-HETE levels being markedly higher in CD than in UC 21, 22. Additionally, lipoxygenases ALOX5 and ALOX15 exert proinflammatory effects, and their genetic inactivation confers protection in dextran sulfate sodium (DSS)-induced colitis models 23. These findings underscore the pivotal role of AA metabolism in sustaining the inflammatory milieu of IBD, a key precursor to CAC.

The role of AA in CRC, particularly CAC, remains controversial, with evidence supporting both anti-tumorigenic and pro-tumorigenic activities. Some studies suggest that AA exerts anti-tumor effects by inhibiting cancer cell proliferation and promoting apoptosis. For instance, AA has been shown to activate neutral sphingomyelinase, increase β2-microglobulin exposure on cell surface membranes for antibody binding, and hydrolyze sphingomyelin to ceramide, a potent inhibitor of proliferation and inducer of apoptosis across various tumor cell lines 24, 25. Furthermore, AA suppresses CRC cell proliferation by disrupting DNA replication and endogenous fatty acid synthesis, primarily through interference with the G1/S cell cycle transition and DNA repair processes, independent of reactive oxygen species production or caspase-3/7 activation 26, 27. In contrast, other studies report that AA induces oxidative damage to DNA and proteins, activates caspase-3/7, and promotes apoptosis, thereby inhibiting CRC cell proliferation 28.

Conversely, substantial evidence supports a pro-tumorigenic role for AA and its metabolites. A Mendelian randomization study by Larsson *et al.* demonstrated a positive correlation between plasma phospholipid AA concentrations and increased risks of colorectal, lung, and esophageal cancers 29. High dietary AA intake leads to the accumulation of prostaglandins, particularly PGE_2_, which fosters a pro-inflammatory microenvironment conducive to cancer development 30. PGE_2_ enhances CRC cell proliferation, migration, and invasion in an autocrine manner and inhibits inflammasome complex formation (ASC/Caspase-1/NLRP3) in THP-1 cells, promoting a shift from pro-inflammatory M1 to pro-tumorigenic M2 macrophages in the presence of AA 31. TXA_2_, another AA metabolite, drives cell growth, migration, and angiogenesis, with elevated levels associated with poor prognosis, reduced survival, and metastatic disease in multiple cancers 32. Overexpression of 5-LOX in the lipoxygenase pathway correlates strongly with risk factors for malignant transformation of adenomatous polyps 33. Additionally, 12S-HETE, secreted by CRC cells, enhances cancer-associated fibroblast growth and angiogenesis, further promoting CRC invasiveness 34.

Chronic inflammation in IBD leads to repeated mucosal injury and repair, increasing the risk of dysplastic transformation and CAC development. Given the dual roles of AA in modulating inflammation and tumorigenesis, elucidating the intrinsic mechanisms of AA metabolism in the inflammatory-to-cancerous transition in CAC is critical. This review synthesizes global and domestic research to clarify the complex interplay of AA and its metabolites in driving CAC, providing a foundation for targeted therapeutic strategies to mitigate disease progression and improve clinical outcomes.

## 4. Mechanisms of Arachidonic acid involvement in inflammatory cancer transformation

**Figure 2:**
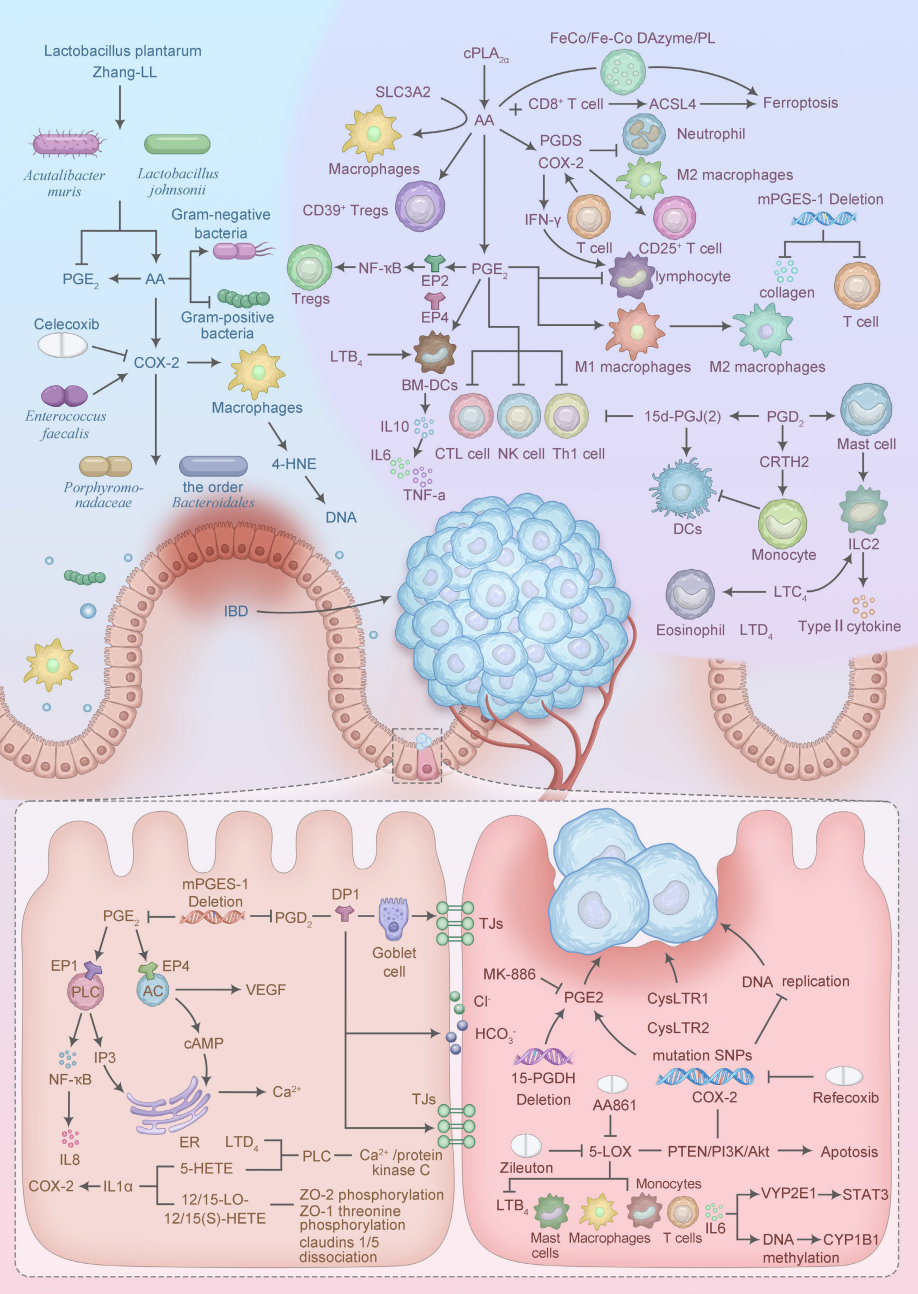
Mechanisms of AA involvement in inflammatory cancer transformation. The blue section in the upper left corner is the intestinal microbiota, the purple section in the upper right corner is the tumor immune microenvironment, the yellow section in the lower left corner is the intestinal barrier, and the pink section in the lower right corner is the Genetic, Epigenetic, and drug. IBD: Inflammatory bowel disease; PGE_2_: prostaglandins E_2;_ AA: Arachidonic acid; COX-2: cyclooxygenase-2; NF-ҡB: Nuclear Factor Kappa-light-chain-enhancer of Activated B cells; LTB_4_: Leukotriene B_4_; IL10: Interleukin-10; IL6: Interleukin-6; TNF-a: Tumor Necrosis Factor-alpha; PGD_2_: prostaglandins D_2;_ DCs: dendritic cells; ILC2: ‌type 2 innate lymphoid cell; LTC_4_: Leukotriene C_4_; LTD_4_: Leukotriene D_4_; IL8: Interleukin-8; ER: Endoplasmic reticulum; TJs: Tight Junctions.

CAC, primarily arising from UC and CD, represents a distinct paradigm of inflammatory cancer transformation driven by chronic intestinal inflammation. AA metabolism underpins this process through tightly regulated molecular mechanisms. cPLA_2_, activated via phosphorylation at Ser505 by inflammatory cytokines (such as IL-1β, TNF-α), selectively hydrolyzes membrane phospholipids to release AA, a process amplified in UC and CD mucosa 35. The liberated AA is metabolized by COX-2, induced by NF-κB, to produce PGE_2_, which binds EP2/EP4 receptors to activate cAMP/PKA and PI3K/AKT pathways, promoting epithelial dysplasia 36. Similarly, 5-LOX, stabilized by FLAP, generates LTB_4_, which engages BLT_1_ receptors to enhance NF-κB and STAT3 signaling, driving immune suppression and tumor progression 37. Novel interventions, such as CRISPR/Cas9-mediated silencing of PLA2G4A, reduce AA availability, attenuating these oncogenic cascades in CAC models 38. These molecular mechanisms link AA metabolism to the subsequent immune, epithelial, microbial, genetic, and epigenetic alterations driving CAC, as detailed below (Figure 2).

### 4.1 Tumor immune microenvironment

The tumor immune microenvironment (TIME) comprises immune cells, fibroblasts, blood vessels, signaling molecules, and the extracellular matrix, all of which shape tumor initiation, progression, and metastasis in CAC. AA metabolism influences the TIME by generating pro-inflammatory and immunosuppressive metabolites, such as PGE_2_ and LTB_4_, which foster a tumor-permissive environment 39. Cytosolic PLA_2_ promotes AA-derived PGE_2_ production, driving lymphocyte infiltration and M1-to-M2 macrophage polarization, which protects the colon from excessive inflammation but promotes tumor tolerance 40. Endogenous lipid mediators, formed via COX-2 and prostaglandin D synthase, reduce neutrophil and M2 macrophage polarization, facilitating IBD remission 41. In trinitrobenzene sulfonic acid (TNBS)-induced colitis mouse models supplemented with AA, T cells increase interferon-gamma (IFN-γ) production in a COX-2-dependent manner, enhancing lymph node cell activation 42. AA also mediates SLC3A2-dependent reprogramming of macrophage phenotypes, promoting M2 differentiation in both *in vitro* and *in vivo* settings 43. Furthermore, the PLA2G4A/AA axis drives CD39^+γδ^ T-regulatory cells (Tregs) polarization, exacerbating tumor progression and metastasis 44.

To counteract these effects, innovative approaches like a multi-enzyme co-expression nanoplatform integrating LOX and PLA_2_ have been developed. This platform ind uces immunogenic ferroptosis—a form of programmed cell death—while upregulating AA expression to enhance ACSL4-mediated tumor cell death, synergizing with CD8^+^ T cell-derived IFN-γ to boost anti-tumor immunity 45. This strategy highlights the potential of targeting AA metabolism to reverse immunosuppression in CAC, offering a bridge to therapeutic interventions. In the *Apc^Min/+^* model of familial adenomatous polyposis, the amount of CD25^+^ Treg increased with elevated COX-2 activity 46. microsomal PGE_2_ synthase 1 (mPGES-1), a terminal synthase that induces the formation of PGE_2_, whose absence in tumors reduces collagen deposition and T-cell exhaustion and regulates the TIME 47.

In general, PGE_2_ promotes acute localized inflammatory responses and phagocyte-mediated immunity in response to the presence of pathogens. PGE_2_-EP2/EP4 signaling has been reported to induce NF-ҡB gene expression to promote inflammation and cause immunosuppression through recruitment and activation of Tregs 48. Selenoproteins in macrophages alleviate inflammation and protect DSS-induced IBD mice by enhancing 15-PGDH-dependent oxidation of PGE_2_ 49. And in the presence of PGE_2_, it promotes IL-10 production by bone marrow-derived DCs (BM-DCs), which in turn down-regulates self-produced IL-6, TNF-a, and promotes immune homeostasis 50. However, high expression of PGE_2_ in tumor tissues suppresses cytotoxic immune responses in CTL, Th1, and NK cells, leading to immunosuppression 51. The PGE_2_ biosynthesis pathway correlates with CD68^+^ macrophage infiltration and CRC tumor progression 52. CAR-T therapy is a novel precision-targeted therapy for the treatment of tumors. PGE_2_ is negatively correlated with memory T cells, and dual blockade of EP2 and EP4 receptors effectively reverses PGE_2_-mediated inhibition of CAR T cells when it is applied to tumor tissues 53.

PGD_2_ promotes type 2 immunity by activating the group 2 innate lymphoid cell (ILC2) to produce type 2 cytokines by affecting the supernatant of mast cells 54. Meanwhile, PGD_2_ inhibits the migration of monocyte-derived DCs through activation of CRTH2 and, together with the metabolite 15-deoxy-Delta(12,14)-PGJ(2) inhibits TH1 cell chemotaxis and reduces IL-12 secreted by TH1 cells 55.

LTs, as an important inflammatory mediator, play a key role in immune responses. The addition of exogenous LTB_4_ promoted the proliferation of BM-DCs in *in vitro* experiments 56. LTD_4_ not only enhances the accumulation and proliferation of ILC2 and promotes the release of IL-5 and IL-13, but also induces increases of eosinophil 57. In addition, LTC_4_ also induces an increase in ILC2 inducing inflammation 58. 5-LOX affects tumor immunity during CRC development and has a pro-tumorigenic role in the immune microenvironment 59. The immunosuppressive TIME shaped by AA metabolites not only promotes tumor growth but also compromises intestinal epithelial integrity, setting the stage for barrier dysfunction.

### 4.2 Intestinal barrier

The maintenance of intestinal epithelial barrier (IEB) function is critical for intestinal homeostasis, and AA metabolites regulate intestinal electrolytes, epithelial cell proliferation, secretion, and tight junction (TJ) integrity. The COX pathway inhibits Cl^-^/HCO_3_^-^ exchange in chromaffin cells, decreasing affinity for Cl^-^ and causing NaCl malabsorption, leading to the development of diarrhea in IBD 60. The secretion of HCO_3_^-^ by the intestinal mucosa is also crucial for preventing acidic digestive damage. Studies have found that PGE_2_ can stimulate the secretion of Cl^-^ and HCO_3_^-^ in the intestines, which has a protective effect on IEB 61. PGD_2_ is able to induce Cl^-^ secretion from the human colonic mucosa by DP1 receptor-mediated means, causing an elevation of cAMP in epithelial cells 62.

In the intestinal mucosal epithelium of IBD patients, increased phospholipid content of AA contributes to the disruption of the intestinal barrier 63. In animal experiments, the expression of AA and its metabolites (19H-PGF1α and 20H-PGF2α) progressively decreases with the decrease of inflammation, suggesting that mucosal healing is regulated by endogenous lipids 64. COX-1 mainly produces endogenous PGs engaged in mucosal protection, while COX-2 mainly produces endogenous PGs engaged in ulcer and intestinal lesion healing. It has been shown that COX-2 expression is significantly elevated in the early stages of CRC development, which further affects epithelial cells by influencing the stromal microenvironment of the tumor 65.

PGs play an important role in maintaining intestinal mucosal integrity, especially PGE_2_. PGE_2_ is involved in stimulating mucus secretion and down-regulating the immune response through EP4 receptors and is protective against ischemic enteritis and DSS-induced colitis. And activation of EP4 receptors promotes healing of intestinal lesions and is associated with up-regulation of VEGF expression and stimulation of angiogenesis 66. In addition, EP4 receptors are involved in colorectal homeostasis and cancer development 67. However, it has also been suggested that PGE_2_ contributes to the redistribution of intracellular calcium concentration and TJ proteins through multiple signaling pathways, including the PLC-IP 3-Ca^2+^ and cAMP-PKA pathways, that induces disruption of IEB function 68. Ptgs2-expressing fibroblasts around intestinal crypts exert paracrine control of tumor-inducing stem cells through the PGE_2_-Ptger4-Yap signaling axis, which helps drive tumorigenesis 69.

Prostaglandin homeostasis in the intestine is critical for maintaining intestinal homeostasis and influencing tumorigenesis. It was found that in DSS-induced mPGES-1^-/-^ mice, this leads to a decrease in PGE_2_ and PGD_2_, resulting in more extensive acute injury affecting recovery. And in DSS-induced* Apc^Min/+^*: *mPGES-1^-/-^* mice, the number of intestinal polyps was reduced 70. Pharmacological studies have found that PGD_2_, through the DP1 receptor, is able to stimulate mucus secretion from goblet cells to reduce intestinal permeability and achieve protection of the IEB 71. Moreover, PGD_2_ promotes the regression of inflammation in the gastrointestinal mucosa 72.

PGE_2_, LTB_4_, and 5-, 12-, and 15-HETE can protect IECs by inducing proliferation and DNA synthesis in IECs 73. BLT2, a receptor for LTB_4_, is expressed only in IECs and epidermal keratinocytes. When BLT2 receptor is overexpressed in IECs it enhances epithelial drug resistance, suggesting that the LTB_4_-BLT2 axis has a barrier function 74. DSS-induced colitis in mice is exacerbated in the absence of BLT2 receptor, which may be correlated with the reduced intestinal barrier function 75. LTD_4_ and 5-HETE alter the proliferation and DNA synthesis of IECs by activating the phospholipase C/Ca^2+^/protein kinase C pathway activation alters paracellular permeability and is involved in IEB disruption, a process that is not dependent on protein kinase A activation by cAMP 76. In addition, LTD_4_ is able to induce proliferation of Caco-2 cells by binding to the cysteinyl leukotriene receptor (CysLTR), which is dependent on PGE_2_ 77.

Tight junctions (TJs) are multiprotein complexes composed of transmembrane proteins with cytoskeletal enclosing rings of actin and myosin, which are important components of the intestinal barrier 78. It was found that 15-HETE regulates IEB permeability and homeostasis through inhibition of adenosine monophosphate-activated protein kinase and increased zonula occludens-1 (ZO-1) expression 79. 12/15-LO-12/15(S)-HETE axis not only stimulates the phosphorylation of ZO-2, but also stimulates the phosphorylation of ZO-1 threonine and the dissociation of claudins 1/5, which mediates the disruption of endothelial TJs and disrupts the barrier function 80. In addition, the COX pathway interacts with the LOX pathway; 5(S)-, 12(R)- and 15(S)-HETEs alone have little effect on COX-2 expression, but they synergize with IL-1α to cause increased COX-2 expression in human colonic myofibroblasts 81.

EETs exhibit anti-inflammatory effects and are elevated in UC patients, with reduced sEH expression in the intestinal mucosa 82. sEH correlates with villin expression, a marker of intestinal cell differentiation 83. Cyp4a14, a cytochrome P450 family member, promotes oxidative stress and exacerbates DSS-induced colitis, while its knockdown protects the colonic mucosa 84. IEB disruption by AA metabolism facilitates microbial dysbiosis, amplifying inflammation and CAC risk.

### 4.3 Intestinal microbiota

The intestinal microbiota maintains immune homeostasis and protects against pathogen invasion, but chronic inflammation disrupts microbial balance, increasing CAC susceptibility 85. AA metabolism interacts bidirectionally with the microbiota. For example, AA supplementation enhances lipid peroxidation by *adherent-invasive Escherichia coli*, exacerbating inflammation in CD patients 86. In another study, AA was found to kill *S. aureus* through a lipid peroxidation mechanism, in which AA is oxidized to reactive electrophiles, which alters S. *aureus* macromolecules and produces toxicity 87.

Clinical studies have found that metabolites such as AA are increased in CRC patients, and the abundance of *Bacteroides fragilis* and *Prevotella* in the bacterial flora is elevated while the abundance of *Blautia* and *Lachnospiracaea* is reduced 88. *ApoE^-/-^* mice not only have disturbed intestinal flora compared to wild-type mice (*Lachnospiraceae_FCS020*, *Ruminococcaceae_UCG-009*, *Acetatifactor*, *Lachnoclostridium*, and *Lactobacillus_gasseri* pathogenic bacteria were significantly increased), their metabolism was also significantly altered (AA metabolic pathways of 20-HETE, PGF2α and LTB_4_ levels were significantly elevated) 89. These results all indicate suggest a close link between gut flora imbalance and AA metabolism.

Lactobacillus plantarum Zhang-LL regulates the activity of *Acutalibacter muris* and *Lactobacillus johnsonii flora*, significantly reduces the expression of PGE_2_, and promotes AA catabolism, which slows down the process of CRC 90. Further studies have found that feeding AA significantly increases the number of Gram-negative bacteria such as *Escherichia coli* and *Enterobacter faecalis*, and decreases the the number of Gram-positive bacteria *Fusarium nucleatum*. The rich microecological environment of Gram-negative bacteria accelerated the conversion of AA to PGE_2_ and promoted tumor growth in AOM/DSS and gut-specific *APC^-/-^* model mice. Notably, the pro-carcinogenic effect of AA was unaffected by the removal of Gram-positive bacteria, whereas the pro-carcinogenic effect of AA completely disappeared after the removal of Gram-negative bacteria. This evidence suggests that AA-regulated intestinal flora promote the development of CRC 91.

COX-2 is also closely related to the regulation of intestinal flora. *Enterococcus faecalis*, a human intestinal commensal, triggers the production of trans-4-hydroxy-2-nonenal (4-HNE) by macrophages via COX-2, which synergistically reinforces the damage of COX-2 to the DNA of the target cells through the bystander effect, leading to the development of CRC 92. COX-2 inhibitors, such as celecoxib, alter intestinal bacteria, such as *Porphyromonadaceae* family and the order *Bacteroidales*, whose metabolites inhibit the development of intestinal polyps in mice 93. Gut microorganisms are also enriched in CYP450, and the solubility of bacterial CYPs, in contrast to the membrane-bound properties of mammalian CYPs, suggests that intestinal bacteria have a great potential to metabolize xenobiotic compounds.

### 4.4 Genetics

Genetic polymorphisms are strongly associated with CAC, and clinical studies have found that the COX-2 -765G > C polymorphism is associated with a reduced risk of CD in the Netherlands and an elevated risk of CRC in Asians, whereas the COX2 8473 T > C polymorphism interacts with NASID and is able to reduce the risk of CRC 94-97. In order to explore the relationship between ALOX5, FLAP, ALOX12 and ALOX15 polymorphisms and CRC risk, a U.S. cohort analysis found that genetic variants in ALOXs may affect the risk of colorectal tumor development and alter the protective effect of NSAID use on CRC 98. Clinical analyses in northeastern China showed that 12-LOX 261Arg > Gln polymorphisms are closely associated with the risk of CRC development and may serve as a potential marker of CRC prognosis 99.

Mutation or deletion of genes is one of the key factors affecting the number and size of tumors. It is generally accepted that COX-2 is overexpressed in tumors and polyps of CRC patients and CRC mouse models and is thought to promote tumor progression. Nevertheless, single nucleotide polymorphisms (SNPs) in the COX-2 gene may alter the function of the enzyme, thereby altering an individual's risk of developing CRC. Based on clinical cohorts, it has been found that carrying the COX-2 Val511Ala SNP is not associated with a risk of CRC, and that the use of NASID in combination can help reduce the risk of CRC in African Americans 100. Animal studies have revealed that mice with mutations in the COX-2 gene significantly reduce the number and size of intestinal polyps 101. The APC gene, a tumor suppressor, is mutated in >80% of sporadic CRC. sporadic CRCs with mutations. When rofecoxib, a COX-2 inhibitor, was used to treat APC mutant mice, the DNA replication rate of their polyps was significantly reduced and was effective in reducing the number and size of intestinal and colonic polyps 102. In the familial adenomatous polyposis (MMR-proficient CRC) *Apc^Min+^* mice and the *Apc^∆716^* mice, COX-2 gene deletion resulted in reduced intestinal tumor formation 103. In addition, *in vivo* and *in vitro* experiments have shown that knockdown of the COX-2 gene inhibits the proliferation and invasion of CRC cells 104. In mouse models of *Apc^Min+^* and AOM, the elevation of endogenous PGE_2_ caused by deletion of the 15-PGDH gene promotes the growth of colonic tumors 105. Interestingly, knockdown of Ptgs-1 and Ptgs-2 (encoding the COX-1 and COX-2 genes, respectively) greatly reduced the number and size of intestinal polyps in *APC^min+^* mice 106.

mPGES-1 and mPGES-2 have been associated with poor prognosis in patients with CRC stages I-III 107. Genetic deletion of mPGES-1 reduces tumor diversity and tumor load in the distal colon, and is significantly protective against carcinogen-induced CRC 108. Compared with mutant APC, tumors with wild-type APC show higher expression of mPGES-1 109. Moreover, mPGES-1 deficiency enhances susceptibility to acute mucosal injury 110. Genetic variants of LOX were found to be one of the risk factors affecting CRC based on a clinical control trial in the U.S., especially the ALOX15 allele variant 111. In sporadic adenomas, genetic variants in the COX1, COX2, and ALOX12/15 genes were found to have a significant impact on CRC in recurrent adenomas 112. Individuals with the ALOX5 VNTR variant genotype are linked to a reduced risk of CRC 98. Additionally, the heterozygous mutant of ALOX12 is only associated with male CRC patients, revealing a gender bias in functional polymorphisms of ALOX12 in relation to CRC patients 113.

The CysLTR (containing CysLTR1 and CysLTR2) is a G-protein eurameric receptor that mediates the action of CysLT. patients with high expression of CysLTR1 and low expression of CysLTR2 have a poorer prognosis 114. Animal experiments showed that AOM/DSS model mice had low-grade atypical hyperplasia of colon polyps and reduced inflammation levels in the Cysltr1^-/-^ group compared with the wild-type group, supporting the important role of CysLTR1 in colon tumorigenesis 115. In addition, based on the biosignature analysis CysLTR2 was positively correlated with immune cell infiltration and immune checkpoints, which could serve as a potential immune target for determining the CRC prognosis as a potential immune target 98.

CYP450 is overexpressed in CRC tissues and cells. Up-regulation of the CYP450 enzyme pathway in CRC plays a crucial role in its pathogenesis and may serve as a new direction for exploring preventive/therapeutic targets in colon cancer. When using pharmacological inhibitors or gene silencing of CYP450 enzymes, AOM/ DSS-induced CRC development can be inhibited 116. Cytochrome P450 1A1 (CYP1A1) enzyme is one of the most important metabolic enzymes responsible for the metabolism of a wide range of xenobiotics 117. Meta-analysis based on the exploration of the relationship between genetic variants and CRC risk revealed that CYP1A1 rs1048943 A > G may increase susceptibility to CRC compared to rs4646903 T > C 118. Overexpression of the CYP24A1 gene in a variety of cancers, including CRC, correlates with tumor invasion, lymph node metastasis, and decreased overall survival 119. Therefore, investigating overexpression or silencing of a single target, or combining it with immunotherapy, may be a useful tool for chemoprevention of CRC proliferation, invasion, and metastasis as a viable option.

### 4.5 Epigenetic

Epigenetic changes, including DNA methylation, histone modifications, chromatin remodeling, and noncoding RNA, are significantly associated with colitis-associated cancer development and progression. The CpG-island methylation pathway (CIMP) is associated with KRAS/BRAF mutations, rewiring of cellular metabolism by two oncogenes, prognosis, and resistance to classical chemotherapy. Patients with high CIMP in CRC have activation of the AA metabolic pathway and exhibit hypermetabolism 120. COX2 methylation in sporadic primary CRC is also closely related to the CpG island methylation phenotype 121. Transcriptional silencing of 15-LOX-1 promotes CRC, and DNA methylation of the 15-LOX-1 promoter is independently of its transcriptional regulation 122. However, the current studies on the association of ALOX15 and CRC epigenetic studies are scarce, and the underlying mechanisms can be further explored subsequently.

Clinical studies have revealed that CysLTR methylation and gene expression profiles are associated with progression, prognosis, and metastasis in patients with CRC 123. Overexpression of IL6 in CRC induces CYP1B1 and CYP2E1 gene expression and alters the metabolic capacity of epithelial cells, with regulation of CYP2E1 expression occurring through a transcriptional mechanism involving STAT3. For CYP1B1 regulation, IL6 downregulates CYP1B1 targeting the microRNA miR27b through a mechanism involving DNA methylation 124. Streptococcus gallolyticus induces CYP1A enzyme activity in an AhR-dependent manner to regulate expression of epithelial cell biotransformation pathways 125.

## 5. Arachidonic acid pathway as a target for drugs that inhibit inflammatory cancer transformation

The AA metabolism enzymes COXs and LOXs and their metabolites (such as, PGs and LTs) have been considered as novel targets for cancer prevention and treatment. Currently, many clinical trials and experimental studies have shown that some Nonsteroidal Anti-inflammatory Drugs (NSAIDs), inhibitors and natural products, etc. inhibit the occurrence and development of CRC by regulating AA metabolism.

### 5.1 Nonsteroidal anti-inflammatory drugs

NSAIDs are common anti-inflammatory drugs with antipyretic, analgesic, and anti-inflammatory effects, and are widely used in cardiovascular and cerebrovascular diseases as well as various types of cancers. There are two main types of NSAIDs, one type is non-selective inhibition of the COX pathway, which includes aspirin, naproxen, ibuprofen, and so on. naproxen, ibuprofen, etc. The other type is a selective COX-2 inhibitor, including celecoxib, refecoxib, etc. Acetylsalicylic acid (aspirin) was the first NSAIDs developed for commercial use in 1897 and was widely used for its anti-inflammatory effects. Epidemiology has found that aspirin reduces mortality and risk of distant metastasis in CRC 126. Experimental studies have shown that aspirin induces apoptosis in enriched Cancer stem-like cells (CSCs), inhibits tumor progression, and enhances the antitumor effects of chemotherapeutic agents. In addition, aspirin directly interacts with p300 in the nucleus, promotes H3K9 acetylation, activates FasL expression, and induces apoptosis in colorectal CSCs 127.

Celecoxib competitively inhibit COX-2, reducing AA conversion to PGH_2_ and subsequent PGE_2_ synthesis, thereby attenuating EP2/EP4-mediated tumor proliferation 128. Indomethacin, a commonly used potent NSAID, inhibits COX enzymes by reducing AA uptake, thereby inhibiting the malignant development of CRC 129. Parecoxib, the only non-enteric administered COX-2 inhibitor among NSAIDs, is able to inhibit epithelial-mesenchymal transition and metastasis of human CRC cells by down-regulation of β-conjugated proteins, and inhibit CRC metastasis in combination with chemotherapeutic agents 130.

The selectivity of NSAIDs for COX-1 and COX-2 actions plays different pharmacological roles depending on their structures, and the effective therapeutic effects of NSAIDs on inflammation stem from the selective inhibition of COX-2 131. Studies have shown that the greater the selectivity of a drug for COX-2 inhibition, the fewer the gastrointestinal side effects it induces, with a good linear relationship. Currently, 1,3-diaryl pyrazole derivatives were found to have significant inhibitory power and sensitivity to COX-2 enzyme and significant anti-inflammatory activity against COX-1 compared to celecoxib and indomethacin, and have dual anti-inflammatory and anti-cancer activity for the treatment of CRC 132.

Clinical trials, epidemiologic and experimental studies have shown that NSAIDs reduce the risk of CRC and mortality and prevent the progression of colitis to CRC. However, the major adverse effects of treatment with NSAIDs lead to gastrointestinal damage (including UC, bleeding, and even perforation) and cardiovascular side effects. Thus, the search for more effective improvements or combinations is an ongoing problem.

### 5.2 Single-target inhibitors

Currently, there are many inhibitors targeting metabolic enzymes or metabolites in the AA pathway, and the inhibitory effects of these inhibitors on CRC are mostly at the stage of experimental studies in animals. mPGES-1 enzyme, a COX downstream enzyme, is a membrane-associated protein with low expression in most tissues, and it can be induced to be produced by proinflammatory cytokines or tumorigenic conditions 133. MK-886, target mPGES-1, a downstream enzyme in the COX pathway, reducing PGE_2_ formation without affecting COX-1-mediated mucosal protection 134.

In addition to COX pathway inhibitors, there are also LOX pathway inhibitors such as Zileuton (5-LOX inhibitor) and PD146176 (15-LOX-1 inhibitor). Zileuton is used to treat asthma patients by inhibiting 5-LOX, blocking the production of LTB_4_ and the BLT_1_-driven inflammatory cascade reaction. Elias Gounaris *et al.* found that *APC^Δ468/+^* mice consuming food containing Zileuton for 12 consecutive weeks showed a decrease in serum LTB_4_ concentration, as well as a significant reduction in tumor-infiltrating mast cells, macrophages, mature monocytes, and pro-inflammatory T-regs at the site of the polyp, which was effective in decreasing the tumors and polyp formation 135. PD146176, a selective 15-LOX-1 inhibitor, significantly inhibited 13-HODE production to promote tumor growth in human CRC HCA-7 cells, while inhibiting 12-HETE production to inhibit tumor growth in mouse CRC MC38 cells 136.

Within the LOX pathway, LTs also have the potential to prevent CRC. A prospective study showed that montelukast targeting the leukotriene pathway by cysteinyl leukotriene receptor antagonist (LTRA) inhibited the formation of ACFs and cell proliferation in IECs, suggesting that LTRA has the potential to prevent CRC 137. In addition, COX-2 inhibitor (NS-398) or 5-LOX inhibitor (AA861) inhibits CRC tumor invasion and proliferation by promoting apoptosis through modulation of the PTEN/PI3K/Akt pathway 138. GSK2256294, an sEH inhibitor, reduces the production of IL2, IL12p70, IL10, and TNFα in IBD patients. Interestingly, GSK2256294 has different potential effects on UC and CD, reducing IL4 and IFNγ levels in the former and IL1β levels in the latter, respectively 139.

### 5.3 Dual Inhibitors

Frequent inhibition of either the COX or LOX pathway results in the conversion of AA metabolism from one to the other, which can lead to serious consequences. COX/LOX inhibitors inhibit both the COX pathway and the LOX pathway, inhibiting the production of their downstream products, improving therapeutic efficiency and reducing adverse effects associated with a single inhibitor. Meanwhile, dual COX/LOX inhibitors provide a safe and effective theoretical basis for the study of new anti-inflammatory drugs. Mukhopadhyay N *et al.* summarized plant-based natural products with dual inhibition of COX/LOX bioactivity in different species, including Tannins, Steroids, Flavonoids, Alkaloids, etc. emphasizing the importance of natural product derivatives 140. Meshram MA *et al.* conducted a review of synthetic bis-COX-2/5-LOX inhibitors covering Thiazoles, 2,3,4-Trisubstituted thiophenes, Pyrazoloquinazolines and others. The design of these novel scaffolds retains the basic structural features of COX-2 and LOX-5 activity while synergizing or enhancing the activity of bis-COX-2/5-LOX, contributing to the discovery of molecules with superior anti-inflammatory activity 141.

sEH is the major epoxide hydrolase involved in the metabolism of EET and is encoded by the EPHX-2 gene on chromosome 8. sEH has been shown to be overexpressed in colitis and CRC 142. Inhibition of sEH on the one hand increases EET to enhance the bioavailability of EET, which has significant anti-inflammatory effects and protective effects on the lungs, heart, gastrointestinal tract, and blood-brain barrier; and on the other hand, it reduces the product DHET, which is involved in monocyte chemoattractant protein-1 (MCP-1)-mediated monocyte chemotaxis 143.

When sEH inhibitors are co-administered with NASID, they are effective in treating cancer and reduce the side effects caused by NASID, and the underlying mechanisms may be related to decreasing monocyte recruitment and inflammation, blocking the endoplasmic reticulum (ER)/mitochondrial stress induced with NASID to reduce epithelial vascular barrier damage, or increase tissue repair and angiogenesis related 144. 4-(5-phenyl-3-{3-[3-(4-trifluoromethyl-phenyl)-ureido]-propyl}-pyrazol-1-yl)-benzenesulfonamide (PTUPB) is a dual COX-2/sEH inhibitor with antitumor activity and organ-protective effects. PTUPB, when used in combination with cisplatin, enhances antitumor properties without increasing toxicity 145. The combination of sEH inhibitors with other drugs is an effective strategy in the transformation of inflammatory cancers, which can be further investigated in clinical trials.

3,3'-Diindolymethane (DIM) is a novel COX1/2 and ERK1/2 inhibitor derived from the derived from indole-3-carbinol found in broccoli and cabbage. In an *in vivo* mouse model, oral administration of DIM inhibits the growth of xenograft colon tumors and can be used in the chemotherapy of CRC 146. Therefore, the design of simultaneous multi-target blockade can effectively overcome the side effects of the drug and suggest new ideas for the development of effective and safe new drugs.

### 5.4 Natural products

Most of the natural products used in the treatment of cancer are derived from plant extracts, and their derived drugs have the advantage of fewer residues and lower side effects (Table 1).

The gut microbiota-metabolite axis may be one of the important mechanisms for the treatment of IBD. The combination of ginseng and Sinensis effectively increases the abundance of beneficial bacteria and decreases the abundance of harmful bacteria through metabolic pathways such as AA metabolism 147. ginsenoside Rk3, a natural anti-inflammatory active ingredient extracted from ginseng, can improve obesity-induced intestinal inflammation by regulating lipid metabolism 148. Protopanaxatriol saponin is also a major active ingredient of ginseng, which can ameliorate pathological damage and reverse abnormal metabolite changes in UC mice through metabolic pathways such as AA 149. Traditional Chinese medicine Clinopodium chinense Kuntze (CC) has anti-inflammatory, antidiarrheal, and hemostatic activities, and it was found that CC can reduce inflammation through the LPS-TLR4-NF-κB-iNOS/COX-2 signaling pathway, and regulate endogenous metabolites such as AA to alleviate UC 150. Jasminum elongatum alleviates UC physiological and pathological symptoms and reverses DSS-induced UC mice via the IκB/p65/COX-2/AA pathway 151. Also, Chrysanthemum polysaccharides ameliorate 2,4,6-trinitrobenzenesulfonic acid (TNBS)/ethanol-induced colitis in rats by adjusting multiple metabolites including AA 152. Animal experiments in which the Pistacia lentiscus oil was administered first, and in which TNBS was given to induce UC, significantly reduced vesiculation and crypt inflammation 153. Acacia saligna butanol extract and its nanoformulation can reduce COX-2, PGE_2_ and IL1β levels, normalize metabolite levels, and ameliorate intestinal mucosal lesions and inflammatory infiltration in UC mice 154.

Some natural products have dual anti-inflammatory and cancer inhibiting activities for both IBD and CRC. natural phenolics, 6-Gingerol (6-G), one of the constituents of Zingiber officinale Roscoe, is able to inhibit ferrometabolism through AA metabolism, exerting anti-inflammatory and antioxidant effects to ameliorate UC 155. 6-G also inhibits the growth of CRC by inhibiting LTA_4_ hydrolase 156. Berberine, an isoquinoline alkaloid, is found in Coptis chinensis and many other plants 157. Berberine has been found to be able to improve serum metabolic homeostasis by inhibiting the AA metabolic pathway and modulating the intestinal microbiome, thereby treating UC 158. In CRC, berberine prevents the growth, migration and invasion of CRC cells *in vitro* and *in vivo* by targeting the COX-2/PGE_2_-JAK2 and STAT3-MMP-2/MMP-9 signaling pathways 159. Moreover, berberine is also able by targeting various pathways, such as the NF-κB/COX-2 pathway, to result in the cell cycle arrest, induction of apoptosis, and inhibition of inflammatory response in CRC cells 160. Emodin, a plant root extract, reduces intestinal inflammation associated with carcinogenesis 161.

Inositol hexakisphosphate (IP6) is a natural phytochemical. Małgorzata Kapral *et al.* found that IP6 prevents CRC by limiting inflammatory events in the colon epithelium by regulating the expression of COX-2 and 5-LOX proteins, as well as by affecting the synthesis and secretion of PGE_2_ and LTB_4_ 162. A natural product, Celastrol, isolated from Tripterygium wilfordii Hook F, can regulate the NF-κB/COX-2 pathway to block the cell cycle and induce apoptosis, and is a potent antitumor inhibitor 163. Products present in some fruits, nuts and vegetables also have anticancer activity, such as ellagic acid, a hydrolyzed metabolite of ellagitannins 164. Umesalma and sudhandiran found that ellagic acid prevented the development of CRC in rats induced by the chemical carcinogen 1,2dimethylhydrazine by targeting the NF-κB/COX-2 pathway 165. lycopene is isolated from tomatoes. Using a mouse xenograft colon cancer model and *in vitro* experiments, Tang *et al.* found that lycopene and fish oil synergistically inhibited COX-2 and PGE_2_, thereby inhibiting CRC development 166.

Chinese herbal formula is considered as one of the common protocols for effective treatment of CAC. Lizhong Decoction (LZD) improves UC by modulating endogenous metabolites such as AA 167. Zhilining Formula (ZLN) repairs the intestinal mucosal barrier and attenuates persistent inflammation in UC mice by modulating AA metabolism 168. Yinhua Miyanling tablets also has a favorable therapeutic effect on UC by ameliorating colonic mucosal damage through multiple endogenous metabolites and AA metabolic pathways, among others 169. Also, Huang lian Jie du decoction (HLJDD) inhibited colonic pathological injury by regulating AA metabolism and alleviated UC in mice 170. Sanwu Baisan Decoction exerts anti-CRC effects by inhibiting the TLR-4/COX-2/PGE-2 pathway, inhibiting the secretion of anti-tumor-promoting immune cytokines, inducing apoptosis of tumor cells, and maintaining intestinal flora 171.

## 6. Conclusions

AA metabolism drives the inflammatory cancer transformation in CAC through eicosanoid-mediated pathways. In CAC, novel insights highlight AA's role in epigenetic regulation, where COX-2 methylation correlates with CpG island methylation phenotypes, promoting KRAS/BRAF-driven oncogenesis. Additionally, 12S-HETE enhances cancer-associated fibroblast activity, fostering tumor invasiveness via stromal remodeling. A pioneering approach involves CRISPR-based ALOX5/15 gene editing, which suppresses LTB_4_ production and inhibits tumor growth in preclinical CAC models, offering a targeted strategy to disrupt pro-tumorigenic inflammation. Furthermore, AA's interaction with the gut microbiota, particularly Gram-negative bacteria, amplifies PGE_2_ production, accelerating CAC progression, while microbiota-modulating agents like berberine counteract this effect by reducing lipid peroxidation. Clinically, serum LTB_4_ and urinary PGE-M levels serve as non-invasive biomarkers for CAC risk stratification. Dual COX/LOX inhibitors, such as licofelone, mitigate compensatory pathway shunting, enhancing therapeutic efficacy with reduced gastrointestinal toxicity compared to NSAIDs. Future research should leverage AI-driven profiling of AA metabolite signatures to guide personalized therapies and explore integration with immune checkpoint inhibitors to boost anti-tumor immunity in CAC. These advancements position AA metabolism as a transformative target for preventing and treating inflammation-driven colorectal cancer.

## Figures and Tables

**Table 1:** Natural products that play a role in inflammatory cancer transformation in colorectal cancer

Ingredients	Origins	Experimental model	Cell lines/animals	Mechanisms	Anticancer/anticarcinogenic effects	References
Ginseng and Sinensis		*In vivo*	DSS-induced mice model	Regulation of metabolic pathways such as arachidonic acid metabolismBeneficial bacteria (such as *Muribaculaceae_norank*, *Lachnospiraceae* and *Akkermansia*)↑Harmful bacteria (such as *Bacteroides*, *Parabacteroides* and *Desulfovibrio*)↓	Improvement of colitis	147
Ginsenoside Rk3	Ginseng	*In vivo*	High-fat diet-induced mice model	PGE_2_, PGD_2_, TXB_2_, HETE, and HODE↓EET and diHOME↑	Improve obesity-induced intestinal inflammation	148
Protopanaxatriol saponin	Ginseng	*In vivo*	DSS-induced mice model	TNF-α, IL-6, and IL-1↓MPO and NO↓	Inhibit metabolic dysfunctionReversing abnormal metabolite changesAmelioration of pathological damage	149
Clinopodium chinense Kuntze		*In vitroIn vivo*	Mouse macrophage RAW264.7 cellDSS-induced mice model	LPS-TLR_4_-NF-κB-iNOS/COX-2 signaling pathwayNO, PGE_2_, IL-6, IL-10 and TNF-ɑ↑	Reduces systemic inflammationRegulates metabolism	150
Jasminum elongatum		*In vivo*	DSS-induced mice model	IκB/p65/COX-2/arachidonic acid pathway	Improvement of UC mice	151
Chrysanthemum polysaccharides		*In vivo*	TNBS/ethanol induced rat model	P-p65, TLR_4_, P-STAT3 and P-JAK2	Improvement of colitis rats	152
Pistacia lentiscus oil		*In vivo*	TNBS-induced rat model	Vesiculitis and cryptoinflammation↓	Protects against intestinal inflammation	153
Acacia saligna butanol extract and its nanoformulation		*In vivo*	Acetic acid-induced mice model	COX-2, PGE_2_ and IL1β↓	Improvement of intestinal mucosal lesions and inflammatory infiltrates	154
6-Gingerol	Zingiber officinale Roscoe	*In vitroIn vivo*	Human CRC cell lines Caco2DSS-induced mice model	Iron load and MDA↓GSSG↓ SOD, GSH↑	Anti-inflammatory, antioxidant	155
		*In vitroIn vivo*	Human CRC cell lines HCT116 cellsXenograft mouse model	LTA_4_ hydrolase↓Proliferation↓	Inhibition of CRC progression	156
Berberine	Coptis chinensis and many other plants		DSS-induced mice modelBBR-induced fecal microbiota transplantation model	AA metabolism pathway↓	Regulates the intestinal microbiomeImproves serum metabolic balance	158
		*In vitroIn vivo*	Human CRC cell lines SW620 and LoVo cellsXenograft mouse model	COX-2/PGE_2_- JAK2/STAT3 signaling pathway↓	Inhibited CRC invasion and metastasis	159
		*In vitro*	Human CRC cell lines SW480 cell	Arrested SW480 cell cycle at G2/M phaseMitochondriamediated intrinsic apoptosis↑Angiogenesis and inflammation markers↓	Chemopreventive effect on CRC	160
Emodin	Rheum officinale	*In vitro*	Human CRC cell lines SW620 and HCT116 cellsAOM/DSS-induced mice model	Inflammatory cell, cytokine and pro-inflammatory enzymes↓CD3^+^ T lymphocytes↑	Inhibits cancer-associated intestinal inflammation and prevents CRC progression	161
Inositol hexapphosphate		*In vitro*	Human CRC cell lines Caco2 cells	COX-2, 5-LOX, PGE_2_ and LTB_4_↓	Prevention of CRC	162
Celastrol	Tripterygium wilfordii Hook F	*In vitro*	Human CRC cell lines HCT116 and SW620	Cell apoptosis↑Cell cycle arrestNF-κB/COX-2 pathway↓	Effective treatment of CRC	163
Ellagic acid	Ellagitannin	*In vivo*	1,2-dimethylhydrazine-induced mice model	NF-κB, COX-2, iNOS, TNF-α and IL-6↓5'-ND, gamma-GT, CEA, AFP, CD, ALP, LDH↓	Chemopreventive effect on CRC	165
Lycopene	Lycopersicum esculentum	*In vitroIn vivo*	Human CRC cell lines HT29 cellsXenograft mouse model	p21(CIP1/WAF1) and p27(Kip1)↑Proliferating cell nuclear antigen, β-catenin, cyclin D1 and c-Myc proteins↓MMP-7, MMP-9, COX-2 and PGE_2_↓	Synergistic fish oil inhibits CRC growth and progression	166
Lizhong Decoction (LZD)	Zingiberis Rhizoma, Radix Ginseng, Rhizoma Atractylodis Macrocephalae and Radix Glycyrrhizae	*In vivo*	DSS-induced mice	Improvement of metabolites in plasma and urine	Ameliorate of DSS-induced colitis mice	167
Zhilining Formula (ZLN)	Andrographis herba, Sophorae flavescentis radix and Aucklandia radix	*In vivo*	DSS-induced mice	MPO, IL1β, TNF-α, IL18↓AHR↑, NF-κBp65 axis↓COX-2↓	Repairing the intestinal mucosal barrierReduce persistent inflammation	168
Yinhua Miyanling tablets	Lonicerae Japonicae Flos, Scu tellariae Barbatae Herba, Pol ygoni Avicularis Herba, Pyrrosiae Folium, Clematis Armandii Caulis, Lophatheri Herba, Plantaginis Semen, Dianthi Herba and Junci Medulla	*In vitroIn vivo*	Human CRC cell lines Caco2 cellDSS-induced mice model	TNF-α, IL-6, iNOS↓MPO, MDA, SOD↓	Improvement of colonic mucosal damage	169
Huang-lian-Jie-du decoction (HLJDD)	Copptidis Rhizoma, Scutellaria Radix, Phelodendri Chinensis Cortex and Gardenia Fructus	*In vivo*	DSS-induced mice model	COX-2, PLA_2_ and 5-LOX↓	Reversing metabolite abnormalitiesAlleviates UC mice	170
Sanwu Baisan Decoction	Badoushuang, Zhebeimu and Jiegeng	*In vitroIn vivo*	Mouse CRC cell line CT26Xenograft mouse model	TLR_4_/COX-2/PGE_2_↓Induces apoptosis	Inhibition of CRC progression	171
